# Gene Therapy for Retinitis Pigmentosa: Current Challenges and New Progress

**DOI:** 10.3390/biom14080903

**Published:** 2024-07-25

**Authors:** Yuchen Liu, Xin Zong, Wenye Cao, Wenxi Zhang, Ningzhi Zhang, Ning Yang

**Affiliations:** Department of Ophthalmology, Renmin Hospital of Wuhan University, Jiefang Road #238, Wuhan 430060, China; liuyuchen02@whu.edu.cn (Y.L.); 2021305233008@whu.edu.cn (X.Z.); caowenye@whu.edu.cn (W.C.); zhangwenxi@whu.edu.cn (W.Z.)

**Keywords:** retinitis pigmentosa, gene editing, CRISPR-Cas systems, mutation, autosomal dominant, X-linked, autosomal recessive

## Abstract

Retinitis pigmentosa (RP) poses a significant threat to eye health worldwide, with prevalence rates of 1 in 5000 worldwide. This genetically diverse retinopathy is characterized by the loss of photoreceptor cells and atrophy of the retinal pigment epithelium. Despite the involvement of more than 3000 mutations across approximately 90 genes in its onset, finding an effective treatment has been challenging for a considerable time. However, advancements in scientific research, especially in gene therapy, are significantly expanding treatment options for this most prevalent inherited eye disease, with the discovery of new compounds, gene-editing techniques, and gene loci offering hope for more effective treatments. Gene therapy, a promising technology, utilizes viral or non-viral vectors to correct genetic defects by either replacing or silencing disease-causing genes, potentially leading to complete recovery. In this review, we primarily focus on the latest applications of gene editing research in RP. We delve into the most prevalent genes associated with RP and discuss advancements in genome-editing strategies currently employed to correct various disease-causing mutations.

## 1. Introduction

### 1.1. Retinitis Pigmentosa

The eye, as the organ of vision, features a complex structure, with the retina being a key component. Retinitis pigmentosa (RP) is the most prevalent inherited eye disease among various serious disorders leading to blindness. With a global prevalence of 1 in 5000, RP affects over 1.5 million patients worldwide, inflicting irreversible damage to visual function and significantly diminishing patients’ quality of life [1,2].

RP manifests as an inherited heterogeneous retinopathy characterized by photoreceptor cell death and retinal pigment epithelial atrophy. Typically, it entails the progressive degeneration of rod photoreceptors, resulting in night blindness, progressive visual field constriction, and peripheral retinal pigment accumulation visible during early-stage fundus examination [3]. Patients with the same genetic mutation may exhibit variations in the age of onset and rate of progression, posing a significant challenge for the diagnosis and treatment of RP. Unfortunately, RP remains incurable, with the underlying mechanisms leading to rod and cone cell death remaining elusive [4].

The retinal pigment epithelium (RPE) constitutes the outermost layer of the retina, providing support and nutrition and eliminating waste products [5]. In the retina, photoreceptors, including rods and cones, serve as the primary sensory cells, with cones responsible for color vision and bright field of view and rods responsible for dim-light vision. The progressive loss of photoreceptors can be attributed to genetic abnormalities that disrupt key genes involved in phototransduction, photoreceptor structure, and cell maintenance pathways. Moreover, dysfunction of the RPE can further degrade photoreceptors [5].

In the pathology of RP, the progressive atrophy of rod photoreceptor cells leads to the secondary death of cones. Affected individuals typically experience night blindness and tunnel vision (narrowing of the visual field), while initially retaining normal or near-normal central vision (e.g., reading vision, facial recognition). However, central vision is eventually lost with cone cell degeneration, occasionally exacerbated by retinal edema (called cystoid macular edema). Typical RP symptoms include retinal bone-spicule pigmentation, blood vessel attenuation, and waxy optic disc pallor [6]. These symptoms, known as the classic triad of RP, exhibit variability in severity, age of onset, and rate of progression, indicating the clinical heterogeneity of the disease.

Statistically, RP affects approximately 1 in 5000 people worldwide, emerging as the most common inherited retinal disease. It is usually bilateral; however, unilateral ocular involvement has also been reported [1]. The severity of RP is correlated with its Mendelian inheritance pattern, with autosomal dominant RP (ADRP) representing the mildest form and X-linked RP (XLRP) the most severe. Notably, Koyanagi et al. highlighted East Asia-specific pathogenic variants as the primary cause of RP in the Japanese population [7]. This indirectly indicates that a significant proportion of RP-causing loci remain unknown.

### 1.2. Gene-Editing Technologies and CRISPR-Cas Systems

Recently, gene-editing technologies have been widely used in treating various diseases; however, few gene-editing therapies have yet been approved for eye diseases such as RP. Genome editing introduces DNA mutations in the form of insertions and/or deletions (indels) or base substitutions in a target sequence, employing various techniques, such as the use of zinc-finger nucleases (ZFNs), transcription activator-like effector nucleases (TALENs), and more recently, the clustered regularly interspaced short palindromic repeats (CRISPR)/CRISPR-associated nuclease 9 (Cas9) system.

ZFNs are synthetic protein chimeras consisting of DNA-bound tandem arrays of Cys2-His2 zinc fingers fused to the catalytic domains of the FokI nuclease. They have been widely investigated for their ability to induce precise site-specific genomic modifications. Because FokI is active only as a dimer, ZFNs act as monomer pairs in opposite directions. Each monomer, typically consisting of 4–6 fingers (each with 30 amino acids), recognizes and binds to a target half-site of 12–18 bp on the opposite DNA strand. The two half-sites of the entire recognition sequence are spaced 5–7 bp apart, allowing them to target a unique location in the human genome. ZFNs are more commonly used in plant gene editing [8].

TALENs are similar to ZFNs, except that their DNA-binding domains consist of tandem arrays of transcription activator-like (TAL) effectors. They induce double-strand breaks (DSBs) in target sequences, triggering a DNA damage response pathway that leads to genome modification [9]. Furthermore, while TALENs are as efficient as ZFNs, they are larger and more difficult to assemble and deliver. However, in recent years, the CRISPR-Cas system has revolutionized gene editing with its simplicity, efficiency, versatility, and affordability. Basic CRISPR-Cas9 editing is performed by the non-specific endonuclease, Cas-9, which is guided to a predetermined genomic locus by a guide RNA that provides a 17–20-nucleotide targeting sequence at its 5′ end.

**Figure 1:**
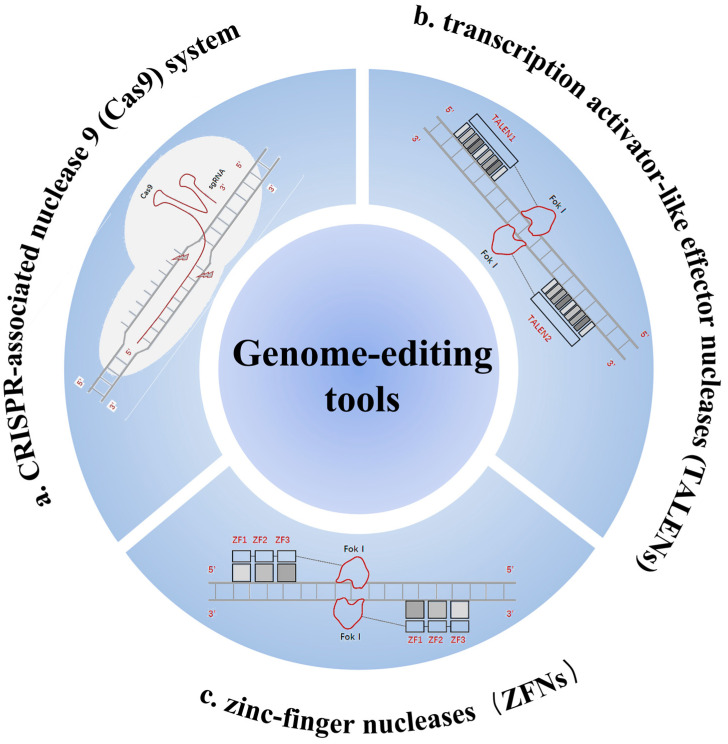
Genome-editing tools. (**a**) The CRISPR/Cas system integrates foreign DNA fragments at regularly spaced short palindromic repeat sites, which are transcribed and cleaved to produce short CRISPR RNAs (CrRNAs). These CrRNAs anneal and bind to trans-activating CrRNA (traCrRNA), guiding the Cas9 protein to mediate sequence-specific degradation of foreign DNA. (**b**) TALENs consist of transcription activator-like effector (TALE) motifs concatenated into a target-determining DNA recognition module, linked to the Folk1 domain. Unlike zinc-finger (ZF) motifs, each TALE motif recognizes a single base pair, creating a one-to-one correspondence with the recognized base pair. (**c**) Zinc finger nucleases (ZFNs) consist of the DNA-binding domain of a ZF protein (ZFP) at the amino terminus and the DNA cleavage domain of a Fok1 endonuclease at the carboxyl terminus. Folk1 is connected to ZFP via the N-terminus and requires dimerization to cleave DNA, necessitating the use of ZFNs in pairs.

Unlike CRISPR-Cas9 systems that target genomic DNA, Cas13 edits RNA for therapeutic purposes, avoiding the risk of causing permanent changes in the genome. A notable development is the compact and high-fidelity Cas13X (hfCas13X), which can degrade targeted RNA with minimal collateral effects and can also be packaged in individual adeno-associated viruses for efficient in vivo delivery and in vitro screening for specifically targeted cells [9]. Although emerging CRISPR-Cas9 gene therapies have advanced the path toward improving vision in patients with RP, the research in this field has encountered several bottlenecks, such as the delivery of the CRISPR-Cas9 complex, the potential risk of immune response, and potential off-target effects [9]. Here is a brief diagram of the three genome-editing tools and their mechanisms (Figure 1).

## 2. Mutation Type and Compatible Therapeutic Approaches

### 2.1. Types of Dominant Mutations

RP can be caused by sporadic mutations, which are the most common cause of the disease; however, genetic susceptibility remains the primary risk factor. In most cases, RP manifests in three different patterns of inheritance: autosomal-dominant (ADRP; 15–25%) [10], autosomal-recessive (ARRP; 5–20%) [11,12], and X-linked (XLRP; 10–15%) [10]. Rarely, dikaryotic and mitochondrial inheritance patterns also occur [13]. In general, different genetic phenotypes of RP have varying prognoses. For instance, XLRP presents with the most severe symptoms and the poorest prognosis [14].

Numerous pathogenic variants in over 80 genes have been identified as causes of RP, complicating diagnosis because some genes exhibit more than one mode of inheritance. In this review, these genes are categorized according to their most likely inheritance patterns. Most genetic variants leading to RP are associated with rod photoreceptors, while a few are linked to the RPE. Various mechanisms lead to rod cell death, including apoptosis, phototoxic or photooxidative damage, endoplasmic reticulum (ER) stress, defective ciliary transport, and defective mRNA processing. The degeneration of optic rod cells alters the retinal environment, subsequently leading to the degeneration of cone cells and the RPE [15].

Mutations can be broadly classified into three types based on the mechanism of causation.

Loss-of-function mutations: These mutations cause the gene product to lose its function, with only one copy of the gene being insufficient to ensure a normal phenotype. This leads to a condition known as haploinsufficiency, which usually results in autosomal recessive disorders [16]. For example, a double mutation in the *RPE65* gene results in *RPE65*-associated RP. Dominant mutations due to haploinsufficiency, though less common, do occur, such as the majority of mutations in *PRPF31*, the pre-mRNA processing factor 31 homologue that leads to ADRP.

Gain-of-function mutations: These mutations confer a new function to a protein that may be toxic to cells. Typical examples include the majority of mutations in the RHO gene, which encodes a photosensitive rhodopsin protein involved in phototransduction. Mutated rhodopsin molecules fail to function properly, and owing to the high levels of rhodospin expression in photoreceptors, a large number of mutated forms may overwhelm cellular mechanisms such as transport or protein degradation [17].

Dominant-negative mutations: These mutations result in a mutant protein that interferes with the function of the wild-type [10]. For example, mutations in the *RP1* gene [18,19] encode a photoreceptor-specific microtubule protein, crucial for membrane disc organization [20,21]. In a knock-in mouse model, altering the ratio of p.Gln662* *RP1* mutant protein to wild-type protein delayed photoreceptor regeneration [22].

### 2.2. Gene Therapy Approaches

Gene therapy involves the alteration of DNA in recipient cells or tissues to achieve desired therapeutic effects. The eye has greater potential for gene therapy than other organs because of the ease of injectable and surgical treatments, immunological advantages, and the ability to assess retinal structure and anatomy using noninvasive techniques [23]. Notably, advanced retinal dystrophy or degeneration is usually irreversible, and treatment success depends on the presence of living retinal cells at the time of initiating gene therapy. Additionally, the single-gene nature of RP has facilitated the development of gene-based therapies.

#### 2.2.1. Therapeutic Strategy

**Figure 2:**
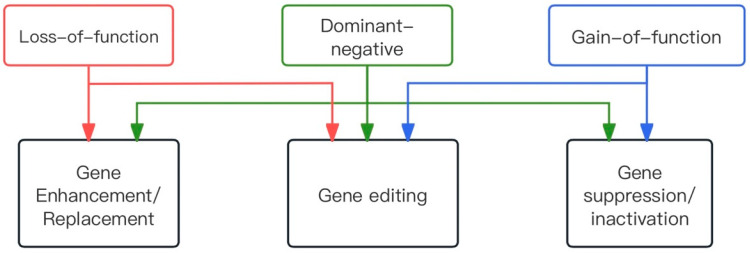
Flow chart showing the appropriate gene therapy based on the type of mutation.

Three corresponding gene therapy strategies were explored based on the type of mutation (Figure 2).

Gene enhancement/replacement: Recessive retinopathy results from a loss of function of the relevant protein, necessitating higher protein levels for treatment. Therefore, supplementing with an additional copy of the wild-type gene may be sufficient to restore the normal phenotype. For example, transgene vectors are introduced into cells using viral vectors or non-viral nanoparticles, with adeno-associated viral (AAV) vectors being the most commonly used because of their high transduction efficiency and excellent safety profile [24]. Additionally, CRISPR activation (CRISPRa) can be employed to enhance gene expression. Notably, complementary genes may not be suitable in cases where cells are sensitive to the protein levels produced.

In some cases of dominant-negative mutations, gene supplementation alone is also recommended [25,26,27]. The rationale is that increasing the ratio of wild-type to mutant proteins will place the mutant proteins at a competitive disadvantage, thereby restoring some degree of function. However, this approach may only alleviate symptoms and not necessarily provide a cure. This is because, even if the mutation primarily manifests as a dominant-negative, it may have toxic side effects on the cells that are not mitigated by the additional inclusion of the wild-type protein [25,28]. Therefore, to treat dominant-negative and gain-of-function mutations, it is generally necessary to disrupt the mutant allele at the DNA or RNA level, followed by genetic enhancement.

Gene suppression/inactivation: Dominant-negative and gain-of-function mutations manifest as altered protein functions. The most direct approach to gene inactivation is to inhibit the production of aberrant proteins. This involves designing molecules that target both mutant and wild-type alleles, thereby blocking protein production before providing an exogenous copy of the gene. An example at the DNA level is transcriptional repression using engineered ZFPs, which have been shown to successfully silence human retinal pigment [29,30]. At the RNA level, ribozymes [31] can disrupt target RNAs, and RNA interference (si/shRNA) techniques [32] can downregulate translation. Another approach is to specifically target mutant alleles while preserving the wild type. At the DNA level, genome editing with the relatively new CRISPR/Cas system has been used to introduce targeted double-stranded breaks for allele-specific ablation [33,34]. Examples of RNA disruption or interference include ribozymes [35] and anti-oligonucleotides [36].

Gene editing: This is an exciting new field in retinal gene therapy. By combining genome-editing techniques with homologous recombination, it is possible to specifically correct mutations at the DNA level. Unlike gene replacement strategies, gene editing permanently reverses genetic defects in target cells. This approach is particularly applicable to autosomal dominant disorders, where gene disruption is more effective than RNA interference. For example, zinc finger nucleases [37] and the CRISPR/Cas system [38] can induce double-strand breaks and facilitate the recombination of donor DNA fragments to directly correct mutant genes in situ. Additionally, base-editing technology provides an efficient method for in situ gene editing.

#### 2.2.2. Viral Vectors

Both viral and non-viral vectors have been evaluated for their efficacy in delivering desirable genes to target cells affected by retinal degeneration. However, viral vectors are the most popular and widely used in therapeutic applications. This is because of their high target cell transduction efficiency, which enables timely transgene expression, as well as their low immunogenicity, which helps evade the host immune response and prolong retention time.

There are three main virus-based vectors: adenovirus (Ad), adeno-associated virus (AAV), and lentivirus (LV) [39]. Among these, AAV is the most promising vector for gene therapy. First described in 1996, AAV is a non-enveloped icosahedral virus with a relatively small size of 25 nm [40]. It exhibits several desirable properties, such as non-pathogenicity, low immunogenicity, and the ability to transduce non-dividing cells and maintain sustained levels of therapeutic gene expression. AAV vectors target both dividing and non-dividing cells, are broadly tropic, and can infect a wide range of cell types [41,42]. AAV serotype 2 (AAV2) is the most commonly used for gene therapy in humans [43]. An AAV-*RPE65* therapy (voretigene neparvovec) was approved by the United States Food and Drug Administration (FDA)(10169 New Hampshire Ave Silver Spring, MD, USA) in 2017 and has now successfully entered phase III clinical trials (CHOP/Spark Therapeutics, Philadelphia, PA, USA; www.sparktx.com (accessed on 10 June 2024)) [21,44]. The intraocular delivery of AAV is well tolerated by the human eye, as supported by data from many ocular gene therapy trials [42,45,46]. Liu et al. demonstrated that early postnatal treatment with AAV vectors containing both the *hRPE65* gene and the Bcl-2L10 anti-apoptotic gene provided enhanced and sustained retinal protection [47]. Many gene therapies using AAV vectors, which are both safe and effective, are also in the preclinical evaluation phase in animal models of inherited retinal degeneration (IRD) [48,49,50,51,52,53]. However, a major drawback of AAV is its small packaging capacity, which prevents it from carrying genes larger than 5 kb, limiting its use for delivering large genes. Scientists are currently addressing this challenge through a new approach that allows the expression of full-length transgenic proteins in transduced cells by splitting the coding sequence into different AAV carriers [54]. This approach has shown that the cloning capacity of AAV can be increased to 14 kb when triple vectors are used [55]. In addition, the use of dual vectors for the treatment of Stargardt disease in Abca4-/-mice was shown to improve the disease phenotype [56].

#### 2.2.3. Tools for Gene Editing

New tools for gene editing have been newly developed to allow effective in situ editing of genes. These tools include Zn-finger (ZF) nuclease, TALE nuclease, Cas9 based on CRISPR-associated RNA bootstrapping, and base editing. Each of these systems is capable of precisely inducing double-strand breaks at specific genomic loci and repairing them via non-homologous end joining, potentially knocking out specific dominant mutant alleles or correcting genetic defects using donor DNA templates via homology-directed repair. However, the effects of off-target mutagenesis and long-term expression of nucleases on terminally differentiated photoreceptor cells need to be carefully assessed before clinical application.

#### 2.2.4. Mode of Administration of Vectors

The vector route of administration also plays a key role in determining the efficiency of gene delivery as well as the type of cell being infected. Subretinal injections and intravitreal injections have been used to deliver gene therapy to the eye, and each method has advantages and disadvantages. Subretinal injection involves injecting the drug into the subretinal space between the photoreceptor cells and the pigment epithelium, which involves separating the neuroepithelial layer of the retina from the pigment epithelium to form the subretinal space. However, subretinal administration requires specialized skills and carries the risk of damaging the delicate retinal tissue, predisposing the retina to detachment and potentially causing glial cell hyperplasia, photoreceptor degeneration, and impairment of visual function. In contrast, injecting drugs into the vitreous cavity via intravitreal injection is easier to administer and less risky than subretinal injections. Delivery of gene therapy drugs into the vitreous cavity of the eye is highly desirable due to its less invasive nature and the ability to distribute the gene drug evenly throughout the retina, potentially allowing for the targeting of more cells. However, vitreous cavity injections may be more likely to cause high levels of inflammation. In addition, because it may generate a humoral immune response against the vector, which, in addition to compromising drug safety, may reduce treatment efficacy and prevent vector expression upon reinjection, it should be avoided if sequential treatment is required in both eyes. In addition, vitreous injections are easily excreted, affecting transfection efficiency. Some vectors are unable to reach the outer retina, including the RPE and choroid, due to the limitation of the inner limiting membrane, and therefore transduction is usually limited to the inner retinal layers [57].

## 3. Genome Editing for ADRP

ADRP accounts for approximately 30–40% of all RP cases, with 38% involving genetic defects that disrupt the splicing pattern [57]. Uncontrolled splicing can lead to various human diseases; however, most splicing variations are associated with ADRP of unknown cause [58]. Over 20 different genes have been identified as causes of ADRP, with only a subset significantly affecting a relevant proportion of patients [58]. The causative genes, categorized as the most common and rare, are described below (Table 1).

### 3.1. Rhodopsin P23H (RHO-P23H)

The most common mutant gene causing ADRP in humans is the RHO gene with a proline-to-histidine substitution at amino acid 23 of the retinal plasma (*RHO-P23H*) [4]. Conversely, retinoschisis mutations represent the predominant cause of ADRP, for which effective treatment or cure remains elusive [15]. However, in a diagnostic and therapeutic study, Wang et al. [63] found that wild-type mice have natural retinal thickness adaptation in early adulthood and observed morphological compensation for photoreceptor degeneration, mainly in the inner nuclear layer (INL) of the retina. Further investigation of the retinal INL in *RhoP23H*/+ mice revealed a negative correlation between the thickness of the INL and outer nuclear layer (ONL). Moreover, electroretinography (ERG) showed an increased b-wave-to-a-wave ratio. These results highlight the continuous morphological events in this model and provide a better understanding of retinal degeneration for future studies.

Cas13X, a new member of the CRISPR family, shows potential as a significant advancement in gene-editing therapeutics. In vitro screening of engineered single-guide RNAs (sgRNAs) specifically targeting *RHO-P23H* mutant transcripts for Cas13x-mediated RNA degradation can tolerate a number of mismatches in sgRNAs without significantly affecting the knockdown efficiency. Yan et al. engineered an sgRNA for *hfCas13X* to specifically knock down the human mutant rhodopsin transcript *RHO-P23H*, achieving targeted knockdown in an ADRP mouse model induced by *RHO-P23H* overexpression. Thus, *hfCas13X* has the potential to treat ADRP caused by RHO mutations and other genetic disorders [10]. Vasudeva et al. [60] observed differences in human clinical lesions based on the type of animal model expressing *P23H* rhodopsin mutants, which poses a challenge in assessing pathogenic mechanisms. Altered autophagy and proteasome activity are considered endogenous mechanisms for clearing misfolded proteins and reducing photoreceptor cell death [4], making them potential targets for the treatment of *P23H*.

New carrier options are also being explored by medical researchers as alternative approaches for gene delivery. Sp et al. investigated the potential of nanoparticle (NP)-mediated delivery of full-length mouse genomic rhodopsin (gRho) or human genomic rhodopsin (gRHO) sequences to counteract the major negative effects of mutant rhodopsin in clinically relevant *P23H*^+/−^ knock-in heterozygous mouse models. This trial showed that mice treated with gRho and gRHO NPs exhibited significant structural and functional restoration of rod photoreceptors, indicating a promising reduction in photoreceptor degeneration [59].

Methods that reduce retinal aggregation in vitro appear to reduce retinal degeneration in mouse models, thereby establishing a possible link between aggregation and retinal degeneration. Additionally, interventions that promote proper folding of mutant retinoschisis, such as enhancing the heat shock response, using partner proteins, or increasing the clearance of misfolded retinoschisis mutants through autophagy or proteasome activity, have all shown beneficial effects in reducing retinal degeneration in mice expressing P23H rhodopsin mutants. Thus, therapeutic strategies targeting the reduction or prevention of retinal protein aggregation may be viable options for combating ADRP [59].

In general, RP remains a significant challenge. The current main approach involves using new methods of gene delivery, such as AAV vectors or nanoparticles, to genetically enhance or inhibit the effect of mutations, thereby enabling cells to restore their original functions. This principle is widely applied in treating mutations at loci, such as P23H and PRPF31, in ADRP. However, the wide range and heterogeneity of mutation loci in ADRP result in a high degree of variability, with many typical loci not yet adequately studied or still being tested in animal models.

### 3.2. Pre-mRNA Processing Factor 31 (PRPF31)

Mutations in the *PRPF31* gene cause ADRP (*PRPF31*-RP), which exhibits incomplete epistasis due to haploinsufficiency, in which reduced levels of gene expression of the mutant allele lead to the disease. Currently, there is no effective treatment for this disease, which makes it a prime target for the development of new therapeutic strategies.

Knowing who will develop RP and who will remain asymptomatic is crucial for affected families. Lisbjerg et al. suggested that the presence or absence of clinical disease in patients carrying the pathogenic *PRPF31* variant is likely related to the expression of the wild-type *PRPF31* allele [64]. This study found no significant differences in the relative quantification of *PRPF31* mRNA expression levels between *RP11* patients and NPC, suggesting that *PRPF3*1 mRNA expression may vary across different tissues and that whole blood is not an optimal medium for monitoring *PRPF31* mRNA expression [64]. The variations in gene expression across different tissues have posed significant challenges in developing a simple clinical assay. Gene sequencing remains the mainstream diagnostic method. In gene therapy, Rodrigues et al. suggested that directly increasing the expression level of *PRPF31* in retinal organoids is effective and sufficient to prevent photoreceptor degeneration [65].

Late-stage *PRPF31*-RP is characterized by permanent photoreceptor loss and significant vision impairment. At this stage, genetic enhancement or genome editing cannot revive the dead photoreceptors. However, alternative approaches have emerged as promising treatments for restoring vision even after photoreceptor loss. Optogenetics involves using photosensitive proteins to stimulate the remaining retinal cells and generate visual signals, while cellular therapies aim to replace the missing retinal cells. Gene-edited cells bypass photoreceptors, stimulating the remaining retinal ganglion cells to send signals to the brain and restore vision. Inducing cells to produce photosensitive proteins through gene-editing techniques, thereby replacing necrotic photoreceptors to transmit light signals, could be a promising future therapeutic direction.

Current research is focused on identifying causative loci and understanding pathogenic mechanisms. Single-allele variants of *PRPF31* are frequently linked to incomplete exanthematous ADRP. Wheway et al. confirmed the pathogenicity of the novel *PRPF31* c.341T>A, p. Ile114Asn variant through in vitro studies. The study showed that computer simulations of the structural effects of missense variants on cryo-EM disassembled protein complexes can help predict the pathogenicity of novel variants when combined with in vitro and clinical studies [66]. Determining the pathogenic status of these missense variants remains challenging. Another research direction involves studying loci and gene editing for targeted corrective therapy; however, research in this area has not yet advanced to therapeutic trials.

### 3.3. Retinitis Pigmentosa 1 (RP1)

Pathogenic variants of the *RP1* gene often lead to the production of truncated proteins [66]. RP1 is known to cause various diseases, including significant superior temporal visual field defects and bony spicules, primarily in the inferior fundus [67]. Patients with RP associated with the m1 variant present with progressive and severe retinal degeneration at an early age [63]. Lázaro-Guevara et al. identified two pure carriers of this variant among three sequenced cases of RP and three heterozygous individuals with adequate *RP1* locus coverage. These findings suggest that combining lineage information with XLC-WGS is a cost-effective method for identifying pathogenic variants [61]. The founder Alu insertion in *RP1* is a significant cause of autosomal recessive RP in Japanese patients and can be overlooked by standard targeted resequencing. Optimized screening for this mutation is recommended, especially for patients with a heterozygous deleterious mutation outside the defined region in exon 4 of RP1 [62].

### 3.4. Cone-Rod Homeobox (CRX)

*CRX* proteins are crucial transcription factors for photoreceptor function and survival. Mutations in the human *CRX* gene are associated with various blinding diseases ranging from mild macular dystrophy to severe conditions like Leber congenital amaurosis (LCA), cone-rod dystrophy (CRD), and RP. These diseases are incurable and mostly inherited in an autosomal dominant manner. Dysfunctional mutant *CRX* proteins exhibit dominant-negative effects by interfering with the function of wild-type *CRX* proteins. Gene enhancement is currently the most promising therapeutic strategy for genetic diseases [4] and could be a potential treatment for *CRX*-associated retinopathy.

Kruczek et al. demonstrated that AAV-mediated *CRX* gene enhancement therapy partially restored the photoreceptor phenotype and phototransduction-related gene expression, as indicated by single-cell RNA sequencing. An induced pluripotent stem cell (iPSC)-derived retinal organoid from a patient with dominant CRX-LCA carrying the *K88N* mutation revealed that deficient optic protein expression is a common phenotype. This deficiency can be alleviated by AAV-mediated *CRX* enhancement in congenital dark haze [68]. Applying similar memory-enhancing therapeutic mechanisms to RP could also provide significant improvements.

## 4. Genome Editing for XLRP

### 4.1. Retinitis Pigmentosa GTPase Regulator (RPGR)

XLRP caused by mutations in the RPGR gene is the most common form of recessive RP, accounting for approximately 70% of all XLRP cases [68,69]. The *RPGR* gene encodes a protein located at the base of the photoreceptor junction cilia, which connects the inner layer of these cells (consisting of organelles that produce proteins and fatty acids required by the cell) to the outer layer (consisting of overlapping discs of photoreceptor cell membranes surrounded by RPE cells responsible for the rhodopsin cycle that leads to vision). This protein is believed to play a role in microtubule organization and ciliary protein transport. Previous studies have identified an interaction network involving *RPGR* and suggested that the *RPGR*-interacting protein (RPGRIP1) aids in the localization of *RPGR* to the attached cilia. When *RPGR* binds to the attached cilia, it regulates intercellular protein transport between the inner and outer segments of the photoreceptors and maintains the correct position and concentration of rhodopsin [70]. However, the exact function of RPGR remains unknown. Pathogenic variants of *RPGR* cause a range of phenotypes, including rod-cone dystrophy (RCD, also known as *RPGR*-associated RP), early-onset severe retinal dystrophy (EOSRD), cone-rod dystrophy CORD, cone dystrophy (COD), macular atrophy, and syndromic XLRP.

Promising positive safety and efficacy results from phase 1/2 clinical trials support the progress of *RPGR* XLRP gene replacement therapy, raising expectations for ongoing and upcoming phase 3 clinical trials that may lead to the long-awaited approval of *RPGR* gene therapy for treatment [70].

Sladen et al. [70] used an established CRISPR/Cas9 gene-editing method to generate isogenic *RPGR* knockout *(RPGR-KO)-iPSCs*. They differentiated *RPGR-KO*, and isogenic wild-type iPSCs were differentiated into retinal organoids to detect AAV-RPGR clinical vector constructs. Transduction of *RPGR-KO* retinal organoids with AAV-*RPGR* successfully restored the expression and localization of RPGR mRNAs and proteins in the connecting cilia of rod and cone photoreceptors. Additionally, AAV-*RPGR* treatment restored rhodopsin localization within *RPGR-KO* retinal organoids and reduced mislocalization to the photoreceptor ONL. These findings provide mechanistic insights into the functional efficacy of *RPGRORF15* gene complementation in human photoreceptors and support the previously reported positive results of a phase I/II trial using this vector construct in patients with *RPGR*-associated XLRP, currently being tested in a phase III clinical trial [69]. However, gene therapy has risks, and its success depends on the efficiency of delivering the therapeutic transgene to the target cell type. Not all gene deliveries are successful, and there are unknown side effects reported during clinical trials. Eighteen patients with genetically confirmed *RPGR* mutations at the Oxford Medical School were recruited into six cohorts of three patients each and were provided increasing concentrations of the codon-optimized (co) AAV2 serotype 8 vector (AAV8.co*RPGR*). As vector concentrations decreased, subjects developed mild inflammation postoperatively, which healed 6 months after hormone therapy [71].

From a deeper molecular perspective, transcriptome studies have shown that the RPGR gene has a complex pattern of optional splicing, resulting in numerous transcripts. The constitutive isoform, *RPGR*ex1–19, is widely expressed throughout the body, whereas the major isoform in the retina, *RPGR ORF15*, utilizes a large open reading frame (ORF15) as the C-terminal exon. All known disease-causing mutations occur in the RPGRORF15 isoform. Using canine and human RNA samples, Appelbaum et al. identified several novel RPGR ORF15-like linear RNA transcripts that contained cryptic introns (exons) in the annotated exon ORF15 [72]. The findings obtained from the novel RPGR circular RNA further emphasize the structural complexity of the *RPGR ORF15* region and offer a potential molecular explanation for the phenotypic heterogeneity of the disease.

Benson et al. [73] retrospectively analyzed a case series of 66 patients with *RPGR*-mediated degeneration. Twelve patients (18.2%) showed retinal sheen on color images, which corresponded to abnormally wide hyper-reflective ellipsoidal bands on optical coherence tomography (OCT) imaging. Three-quarters of these patients were male, exhibited CRD, and harbored *RPGR* mutations at the 3′-end of ORF15 [72,74]. The clinical manifestation of these features may depend on the degree of retinal degeneration and disease progression. Changes in the appearance of abnormal retinal outer bands observed in OCT images after prolonged dark adaptation increased the likelihood of relative phototransduction loss activation [75]. More specific criteria for the diagnosis and treatment of *RPGR*-related conditions are still being explored, but this study provides new insights into the link between morphological changes and their pathological implications.

### 4.2. RP2

Pathogenic variants of *RPGR* and *RP2* are the leading causes of XLRP, accounting for 80–90% of cases. Approximately 15% of XLRP cases result from mutations in *RP2*, potentially leading to a protein truncation affecting protein transportation to the cell surface and Golgi structure maintenance. However, gene therapy targeting this gene is challenging [73], and most carriers are asymptomatic, displaying subclinical features such as temporal lobe reflex (TLR) and pigmentary changes [76]. *RP2*-related retinopathy predominantly manifests in childhood, characterized by rapid progressive retinal degeneration, macular involvement, and early complete loss of the ellipsoid zone [77]. Unfortunately, recent studies have primarily focused on clinical cases and specific *RP2* phenotypic symptoms over the past five years, with targeted gene-editing therapies yet to be developed.

This article summarizes the clinical research on XLRP as follows (Table 2).

## 5. Genome Editing for ARRP

ARRP accounts for 50–60% of all RP cases, with genetics being the primary cause of autosomal recessive disorders. While more than 40 genes are associated with ARRP, only a select few contribute to a significant proportion of cases, as described below (Table 3).

### 5.1. Phosphodiesterase 6 (PDE6) Gene Complex

The *PDE6* complex regulates the concentration of cyclic guanosine monophosphate (cGMP) in the photoreceptor cytoplasm and plays a crucial role in the visual phototransduction cascade. The rod photoreceptor cGMP phosphodiesterase consists of four subunits: the catalytic α-subunit (*PDE6A*), the catalytic β-subunit (*PDE6B*), and two inhibitory γ-subunits (*PDE6G*). Variations in the rod-specific *PDE6* gene family cause ARRP in 8% of cases [78], with variations in genes encoding the *PDE6A* and *PDE6B* subunits being the main contributing factors [79].

In the visual phototransduction cascade in optic rod cells, light excitation of retinal activating transducer proteins leads to the release of the inhibitory subunit of the *PDE6* complex. This induces the activation of the catalytic subunits of the *PDE6* complex (*PDE6α* and *β*), which then hydrolyze cGMP, leading to membrane hyperpolarization and the conversion of light into nerve impulses. This leads to the permanent opening of cation channels in rod photoreceptor membranes (cGMP control), allowing an influx of excess extracellular ions into the cell, eventually culminating in rod cell apoptosis [80].

#### 5.1.1. *Phosphodiesterase 6A (PDE6A)*

The (*PDE6A* gene, located at position 5q32, spans a length of 87 kb and consists of 22 exons, encoding the 860-amino acid alpha subunit of the *PDE6* complex [81]. It is the 7th locus identified as contributing to RP and is implicated in 4% of all cases of ARRP, particularly in severe forms [82,83]. The *PDE6A* variant associated with ARRP was first identified in 1995 [82], and to date, 40 pathogenic variants of this gene have been reported, with the majority (65%) being point variants. In North America, the *PDE6A* gene accounts for 3–4% of all ARRP cases, while rare cases of *PDE6A*-associated RP have been identified in Spanish, Japanese, and UK populations. Meanwhile, ARRP-induced *PDE6A* variants contribute to 2%, 2%, 1.6%, and 1% of cases in the Pakistani, French, German, and Israeli populations, respectively [83].

Although mutations in the *PDE6A* gene have been identified as a cause of human RP, definitive therapies for treating this blinding disease are yet to be developed. Mowat et al. conducted a retinal gene amplification assay in *Pde6a*-mutant dogs, using serotype 8 capsid mutant AAV to deliver Pde6a. This study marked the first successful gene amplification of *Pde6a* RP in a large animal model (Table 3) [84]. In a preclinical gene supplementation trial, administering the rod-specific transgene *AAV2/8(Y733F)-Rho-Pde6α* prior to the onset of the disease in mice resulted in a more effective rescue of photoreceptor cells [85]. Thus, it has been proposed that the success of therapeutic clinical trials will depend on the ability to identify patients early enough to maximize the number of viable rod cells that can be treated with gene therapy [73].

**Table 3:** Clinical research on ARRP.

Target Gene	Type of Method	Outcome	Sample Size	References
*PDE6A*	Serotype 8 capsid mutant adeno-associated viruses was used to deliver *Pde6a*	First successful amplification of *Pde6a* in a large animal model of RP	10	[83]
	Whole mount analysis Immunoblot analysis Electroretinograms	AAV2/8(Y733F)-Rho-Pde6α is an effective gene therapy for mid-stage patients with partial peripheral vision loss	(-) *	[85]
*PDE6B*	*Pde6b*-associated RP mouse retinas were edited in vivo using a dual AAV system with PESpRY through a non-NGG PAM (GTG)	Vision loss from RP-related gene mutations can be prevented by unconstrained in vivo prime editing in degenerating retinas. Family history of affected females with RP does not exclude X-linked disease	(-) *	[86]
*USH2A*	Locus-specific RNA-Cas9 ribonucleoproteins were used with subsequent homologous recombination repair induced by an engineered template supply	Successful in vitro mutation repair	(-) *	[87,88]
*RPE65*	Subretinal injections of the optimized dual-AAV split-PE3 were administered to rd12 mice with the inherited retinal disease Leber congenital amaurosis	Prime editors corrected the pathogenic mutation with up to 16% efficiency, precisely restoring *RPE65* expression, rescuing retinal and visual function, and preserving photoceptors, without detectable off-target edits	(-) *	[89,90]

* (-) means sample size is not mentioned in this reference.

#### 5.1.2. *Phosphodiesterase 6B (PDE6B)*

The *PDE6B* gene, encoding the β-subunit of the *PDE6* complex, is located at 4p16.3, spanning a total length of 45 Mb. It comprises 22 exons and encodes an 854-amino acid protein [91,92]. This gene was the first to be identified as causing ARRP. The ARRP-associated variant was first identified in 1993. RP resulting from *PDE6B* mutations typically presents aggressively, with early onset severe vision loss due to rapid photoreceptor degeneration. Genetic defects in this gene account for approximately 5–8% of ARRP cases [92].

Numerous studies have explored *PDE6b*-related animal models. Yeo et al. suggested that *Pde6b-KO* rats exhibit photoreceptor degeneration with a slower disease progression and larger anatomical structures compared to the corresponding mouse model. Consequently, they may offer a more suitable model for experimental therapies targeting RP [81]. Yang et al. described the development of a novel *Pde6b-KO* Long-Evans rat model mirroring key features of human RP. This model can be utilized in preclinical translational studies to further investigate retinal degeneration [93].

In terms of gene therapy, Bitoque et al. tested the combination of a previously developed non-viral vector [94] with a plasmid optimized for the sustained expression of *PDE6β* using the rd10 mouse model. Kuehlewein et al. investigated the phenotypic and genotypic characteristics of RP associated with the *PDE6B* variants. Genetically, 43% of the patients harbored de novo *PDE6B* variants, significantly contributing to the spectrum of *PDE6B* variants. Additionally, 83% of the patients exhibited less severe visual impairment, offering a window of opportunity to investigate new drugs [95]. Han et al. described the production of two different AAV vectors (AAV2/1 and AAV2/5) manufactured under current Good Manufacturing Practice standards and demonstrated their ability to induce human *PDE6B* expression in vivo. AAV-mediated subretinal gene therapy delays photoreceptor cell loss in Pde6b-deficient rats compared to untreated controls [86]. Qin et al. developed a genome-editing tool, PESpRY, characterized by the versatility of the prime editor (PE) and the unconstrained protospacer-adjacent motif (PAM) requirement for SpCas9 variants (SPRY). They utilized a dual-AAV system to transduce the *Pde6b*-associated RP mouse model with PESpRY for in vivo genome editing, targeting a non-NGG PAM. The treated mice exhibited significant electroretinogram responses, enhanced visual stimulus-driven optomotor responses, and proficiency in a visually guided water maze task. This underscores the potential of unrestricted in vivo genome editing in degenerating retinas to prevent vision loss associated with mutations in RP-related genes [96].

### 5.2. USH2A

The *USH2A* gene, encoding a guidance protein, is located at 1q41. It spans a length of 800 kb on genomic DNA and comprises 73 exons (with exon 71 being cochlear specific). Mutations in *USH2A* represent the primary cause of ARRP, accounting for 12–25% of cases. This protein plays an integral role in the development of cochlear hair cells and maintenance of photoreceptors. It is expressed in the retina, especially in the inner part of the photoreceptors, as well as in the supportive tissues of the inner ear [97].

Genetic variations in the *USH2A* gene result in two distinct phenotypes: non-syndromic RP and Usher syndrome type 2a. Over 1100 pathogenic variants of USH2A have been identified, including missense, nonsense, splice, deletion, insertion, deletion, and major rearrangements [98]. Among these variants, insertion mutations lead to the most severe forms of ARRP, followed by splice and missense variants [97].

At the molecular level, the pathogenesis of *USH2A* has not been fully elucidated. However, studies involving *USH2A* cells and animal models generated using human iPSCs (hiPSCs) or CRISPR/Cas9 technology will help further unravel the pathogenesis of *USH2A* [99]. Extracting hiPSCs from patients with *USH2A*-associated diseases provides an opportunity to dissect the underlying molecular mechanisms and explore potential innovative therapies. A pioneering study by Tucker et al. (2013) utilized a 3D/2D protocol to derive hiPSCs from a patient carrying the c.7595-2144 A > G mutation (rs786200928) in USH2A intron 40 and the trans c.12575G > A mutation (rs199605265) in keratinocyte cells of a patient from whom ocular capsular structures were generated. While USH2A-derived retinal cells showed no significant differences in early developmental abnormalities compared to controls, they exhibited elevated levels of GRP78 and GRP94 proteins, indicating potential involvement of ER stress in *USH2A*-related pathogenesis [100]. Liu et al. characterized an Ush2a-deficient mouse model, revealing complete loss of the two usherin isoenzymes in the retina and cochlea. A subsequent study based on this model showed that retinal degeneration is late-onset and progressive [101]. The Usher-like mouse model of KMUSH/USH exhibits spontaneous RP and moderate hearing loss, along with reduced expression of both Pde6b and Ush2a (Yao et al., 2016). Several point mutations have been identified in Ush2a, suggesting its pathogenic role in the KMUSH/USH usher-like phenotype [102].

Currently, there are no approved treatments for RP caused by *USH2A* mutations; however, related research is ongoing. Viral gene therapy using AAV has emerged as the safest and most effective method of retinal cell transduction to date. However, with a load limit of only 4.7 kb, individual AAV particles are incapable of carrying the complete *USH2A* coding sequence (15.6 kb). To overcome this limitation, several strategies have been explored and optimized. The utilization of double and triple AAV systems has expanded the translocation capacity from 4.7 kb to a maximum of 14 kb. Maddalena et al. employed intron-mediated protein transduction to enhance the translocation capacity of AAV into the retina. However, the practical application of intron-mediated protein transduction through AAV vectors is constrained by the necessity of incorporating cis-regulatory sequences into each AAV, thereby limiting its versatility [55]. Toms et al. successfully generated scaffold/matrix attachment region (S/MAR) DNA plasmids containing the full-length human *USH2A* coding sequence, a GFP reporter gene, and a ubiquitous promoter (CMV or CAG). This was the first report of a vector capable of expressing full-length usherin and performing functional rescue [87].

Advancements in CRISPR/Cas9 technology have streamlined the application of gene-editing strategies to rectify point mutations and small chimeras, offering promising strategies for the treatment of inherited diseases. Gene editing enables the correction of *USH2A* mutations directly within the patient’s retina and holds potential for correcting hiPSC-derived photoreceptors for future transplantation purposes. Thus far, CRISPR/Cas9 targeting of *USH2A* has involved correcting the recurrent c.2299delG, p. Glu767Serfs*21 mutation (rs80338903), and c.2276 G > T, p. Cys759Phe (rs80338902) in an HEK cell model, as well as patient-derived fibroblasts and hiPSCs being edited in vitro [88,103]. Although gene-editing therapies have shown proof-of-concept success in vitro, numerous challenges remain in terms of clinical translation.

### 5.3. Retinal Pigment Epithelium 65 (RPE65) Gene

The human *RPE65* gene, located at 1p31, has a nucleotide length of 20 kb and consists of 14 exons. It encodes a highly conserved RPE-specific 65 kDa protein, which has two isoforms: membrane-bound mRPE65 and soluble sRPE65 [72]. It plays a pivotal role in the visual cycle [104], a series of biochemical reactions involving retinol in ocular tissues supporting vision in vertebrates. Upon light exposure to photosensitive pigments in the retina, 11-cis-retinal is converted into all-trans retinyl esters. *RPE65* acts as an isomerase that reconverts all-trans retinyl esters into 11-cis-retinal. Double-allele mutations in the *RPE65* gene can reduce or eliminate *RPE65* activity, disrupting optic circulation and leading to visual impairment.

Genetic defects in RPE cells contribute to 5% of RP cases, with the *RPE65* gene accounting for half of these cases [105]. Mutations in RPE65 induce photoreceptor degeneration, leading to RP (predominantly ARRP and occasionally ADRP) and LCA. RPE65-related inherited retinal diseases typically manifest between birth and five years of age, with patients experiencing complete vision loss by their fourth decade of life (legal blindness). Over 300 variants of the *RPE65* gene have been identified, most of which are point variants.

Currently, Luxturna (voretigene neparvovec) is the only gene therapy licensed for the treatment of RP. Luxturna delivers a functional *RPE65* gene copy via an AAV into retinal cells through a subretinal injection, facilitating normal *RPE65* expression in retinal cells. However, it is licensed and authorized to treat only a small proportion of patients with mutations in *RPE65*. This selected group, necessitating double mutations and viable photoreceptor cells, constitutes merely 0.3–1% of all RP cases. In a phase III clinical trial, 31 patients diagnosed with bilateral *RPE65* gene mutations were treated with voretigene neparvovec, an AAV2 vector carrying a modified human *RPE65* gene. This intervention led to a significant improvement in visual function after one year, which was sustained over three to four years of follow-up, with no notable adverse effects reported [106,107,108]. Deng C et al. demonstrated the real-world safety and efficacy of voretigene neparvovec gene therapy in pediatric patients with a two-copy *RPE65* pathogenic variant [109]. However, the success of the *RPE65* gene therapy in long-term progression halting is limited to retinal areas where photoreceptors remain relatively intact at the time of intervention. Additionally, there may exist unknown mechanisms contributing to only partial therapeutic effects beyond the subretinal injection site [110]. Hull et al. performed clinical examinations, retinal imaging, and electrophysiological tests on four patients from four families affected by early-onset retinal dystrophy. They performed bidirectional Sanger sequencing for all exons and intron–exon boundaries of the *RPE65* gene. Their findings indicated that low-ploidy variants in *RPE65* are associated with mild childhood disease and preserved vision in adulthood, suggesting that transduction efficiency may not be the sole limiting factor for enhancing vision in gene replacement therapy trials. Instead, achieving optimal vision recovery may necessitate the administration of gene replacement therapy earlier in childhood [89].

Furthermore, PEs can edit a wide array of genomes, including all 12 possible base substitutions, along with a limited number of insertions, deletions, and combinations thereof, without the need for double-stranded breaks or exogenous donor templates. This versatility of PEs in correcting disease-causing mutations is expected to expand the therapeutic potential of gene editing. She et al. investigated the application of PEs in a study involving a patient afflicted with the hereditary retinal disease LCA. Upon administering subretinal injections to rd12 mice with the same hereditary retinal condition, they observed the primary editors correcting disease-causing mutations with significant precision, achieving an efficiency of up to 16%, with no evidence of off-target editing. This intervention resulted in the restoration of *RPE65* expression, rescue of retinal and visual functions, and preservation of photoreceptors. These findings have spurred further exploration of PE for treating inherited retinal diseases resulting from various mutations [90].

## 6. Conclusions/Outlook

With research advancements and knowledge dissemination, the field of RP is undergoing a significant paradigm shift. In particular, the rapid progress in gene therapy has led to positive stage-by-stage experimental outcomes in translational applications for RP therapy. This article provides an overview of the latest advancements in gene editing research for RP, highlighting the current progress in genome-editing strategies aimed at correcting various disease-causing mutations, including clinical trials (Table 4). Gene therapy, being relatively safer and less invasive in the short term compared to other treatment modalities, has emerged as a more effective option. Innovations in gene therapy technology have enabled the modification of disease-causing genes, paving the way for personalized and precise RP treatments. Although gene-editing therapies have shown proof-of-concept for many RPs caused by gene mutations in vitro, significant challenges remain in terms of clinical translation. Currently, Luxturna (voretigene neparvovec) is the only approved gene therapy for RP treatment; however, it is limited to a small subset of patients with mutations in the *RPE65* gene. Moreover, like other therapies, the efficacy, safety, and stability of gene editing require further improvement. Nonetheless, ongoing research efforts are showing promising results, suggesting gene editing as a potentially effective therapy for RP.

## Figures and Tables

**Table 1:** Clinical research on ADRP.

Target Gene	Type of Method	Outcome	Sample Size	References
*RhoP23H*, *RhoG188R*	Western blotting	Beneficial effects in reducing retinal degeneration in mice expressing P23H rhodopsin mutants	(-) *	[59]
*PRPF31*	Elucidated structure of the intact spliceosome to model the effect of a novel PRPF31 variant	Whole blood is not the optimal medium for monitoring PRPF31 mRNA expression	176	[60]
*RP1*	XLC-WGS	Combining lineage information XLC-WGS is a cost-effective way to identify pathogenic variants	17	[61]
	The Alu insertion in *RP1* was screened with an optimized PCR-based method	The founder Alu insertion in *RP1* is an important cause of autosomal recessive RP in Japanese patients and can be overlooked in standard targeted resequencing.	26	[62]

* (-) means sample size is not mentioned in this reference.

**Table 2:** Clinical research on XLRP.

Target Gene	Type of Method	Outcome	Sample Size	References
*RPGR*	Optical coherence tomography (OCT)	Three-quarters of patients were male, exhibited cone-rod dystrophy	66	[74]
	AAV8.co*RPGR*	data Data as vector concentrations decreased, subjects developed mild inflammation postoperatively but healed 6 months after hormone therapy	18	[71]
*RP2*	OCT and electrophysiology	Most carriers were asymptomatic, exhibiting subclinical characteristics, female carriers of *RP2* variants can manifest RP, family history of affected females with RP does not exclude X-linked disease	40	[77]

**Table 4:** Summary of RP clinical trials.

Genotype	Target Gene	Genome-Editing Tool	Disease Application	Outcome	References
ADRP	*RHO-P23H*	hfCas9X-based sgRNAs	Toxic rhodopsin	Protective role of hfCas13X-mediated targeting in photoreceptor degeneration	[9]
	*P23H* ^(+/−)^	Rho and gRHO nanoparticle	Photoreceptor degeneration	Promising reduction of photoreceptor degeneration	[111]
	*PRPF31*	CRISPR/Cas9	Prevention of photoreceptor degeneration	Directly increasing PRPF31 expression in retinal organoids effectively prevents photoreceptor degeneration. K88N mutation can be alleviated by AAV-mediated CRX	[64]
	*CRX-LCA*, *CRX-K88N*	scRNA-seq, AAV	Determination of disease-associated changes in photoreceptor subtypes and evaluation of treatment effect following gene therapy	K88N mutation can be alleviated by AAV-mediated CRX	[112]
X-RP	*RPGR*-KO	data CRISPR/Cas9, AAV-RPGR	Successful restoration of expression and localization of RPGR mRNAs and proteins connecting cilia in rod and cone photoreceptors	Photoreceptor degeneration	[69]
ARRP	*RPE65*	AAV2-hRPE65v2	Improvement and maintenance of functional vision after administration of voretigene neparvovec (VN; Luxturna [Spark Therapeutics, Inc.]) in patients with biallelic *RPE65* mutation-associated inherited retinal disease	Significant improvement in visual function without serious adverse effects after one year, sustained over three to four years of follow-up	[105,106,107]

## Data Availability

No new data were created or analyzed in this study. Data sharing is not applicable to this article.
